# Unveiling the significance of prokaryotic composition from ferromanganese crusts regarding the interlink between cobalt and vitamin B_12_ in deep-sea ecosystems

**DOI:** 10.3389/fmicb.2025.1524057

**Published:** 2025-04-29

**Authors:** Lilia Montoya, Elva Escobar-Briones

**Affiliations:** ^1^Consejo Nacional de Humanidades, Ciencias y Tecnologías, Mexico City, Mexico; ^2^Instituto de Ciencias del Mar y Limnología, Universidad Nacional Autonoma de Mexico, Mexico City, Mexico

**Keywords:** food web, meiofauna, prokaryotes, biogeochemical cycling, auxotrophy

## Abstract

The intricate relationship between prokaryotic vitamin B_12_ (cobalamin) producers and metazoans in deep-sea ecosystems, particularly within ferromanganese crusts and polymetallic nodules, is critical for understanding oceanic biogeochemical cycling of cobalt. Microbial communities are key regulators of essential biogeochemical cycles, with cobalt serving as a vital component in the synthesis of cobalamin, a metallocofactor indispensable for numerous metabolic processes. We analyzed the significance of cobalamin biosynthetic pathways confined to prokaryotes and emphasized the ecological importance of auxotrophic organisms that rely on exogenous sources of vitamin B_12_. Additionally, we recognize recent research regarding the spatial distribution of dissolved cobalt and its consequential effects on cobalamin production and bioavailability, indicating the scarcity of cobalt and cobalamin in marine environments. We propose that cobalt-rich environments may foster unique interactions between prokaryotic and eukaryotic organisms, potentially altering the food web dynamics owing to the localized abundance of this element. By investigating the roles of cobalt and cobalamin in nutrient cycling and interspecies interactions, we outlined key criteria for future research on deep-sea microbial communities and their contributions to the cobalt biogeochemical cycle.

## 1 Introduction

Microbial communities represent most of the ocean’s diversity and are of fundamental importance in maintaining the functionality and stability of global ocean ecosystems. Microorganisms, while not strictly essential for survival, play a pivotal role in regulating marine C, N, P, and S biogeochemical cycles. However, trace elements (<0.1 μM) are also required for growth and are interlinked with the global biogeochemical cycles of macro and microelements (Giovannelli, 2023). One such example is cobalt (Co), as cobalt and iron cycles are co-regulated by phytoplankton, as they require both nutrients for optimal growth, and the availability of one can influence the utilization of the other (Chmiel et al., 2022).

In addition, Co is used for numerous metabolic functions, including non-corrin Co-containing enzymes such as methionine aminopeptidase, prolidase, nitrile hydratase, glucose isomerase, methylmalonyl-CoA carboxytransferase, aldehyde decarbonylase, lysine-2,3-aminomutase, and bromoperoxidase (Okamoto and Eltis, 2011).

We focused on cobalt as a central constituent of the corrin cofactor cobalamin or vitamin B_12_ present in early life as methanogenesis (Buan, 2018) and globally as phytoplankton, particularly cyanobacteria, which have an absolute requirement for cobalamin (Chmiel et al., 2022). Cobalamin has been demonstrated to be of crucial importance in trophic networks from several environments, including marine systems. Consequently, Co can limit certain metabolic pathways, potentially restricting the metabolic niches of cobalamin synthesis and auxotrophy, and incentivizing ecological relationships linked to the acquisition of vitamin B_12_, such as predation and symbiosis.

The sensitivity of trophic networks to Co has been studied in Co-depleted systems with nutrient amendments. In this regard, we propose to study sites naturally characterized by a high abundance of cobalt. We specifically refer to ferromanganese nodules and cobalt-rich crusts, which are mineral deposits found on the deep ocean floor that are enriched in metals, such as manganese, iron, copper, nickel, and cobalt. These mineral concretions are recognized as important habitats that support a unique deep-sea biodiversity (Rabone et al., 2023).

We review the importance of understanding the sources and delivery pathways of vitamin B_12_ in marine ecosystems, particularly those linked to polymetallic nodules and cobalt crusts, considering that the bioavailability of cobalt may regulate its use in different life forms. This allowed us to gain new insights into the biogeochemical cycling of Co in deep-sea ecosystems.

## 2 Cobalamin: structure, biosynthesis and ecological significance

### 2.1 Cobalamin is a corrinoid with cobalt

**Figure 1:**
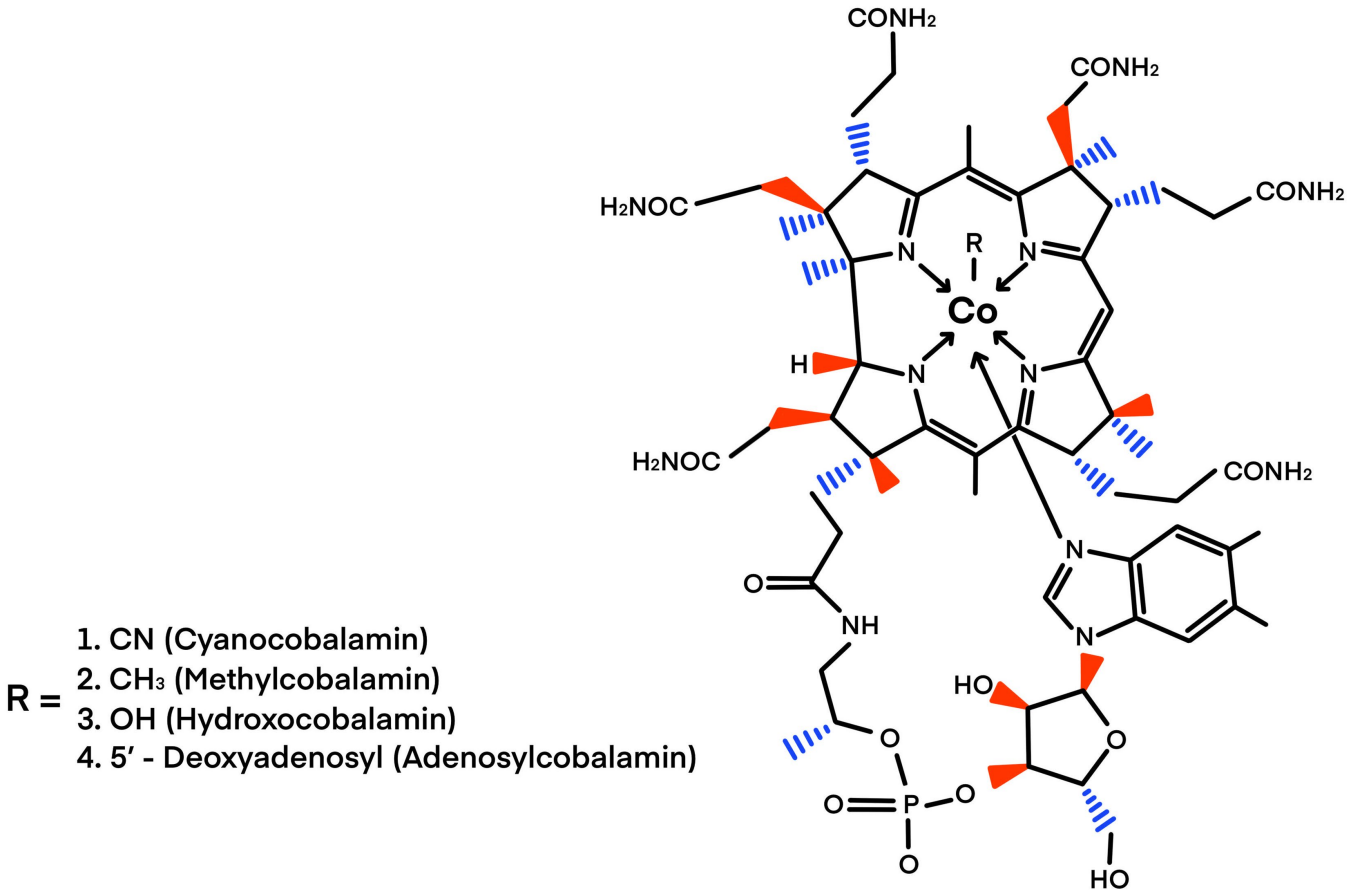
Structure of cobalamin. The cobalt ion is coordinated equally to the four pyrrolic nitrogen atoms of the corrin macrocycle. Cobalamines differ in their sixth ligand (R −). The sixth ligand differentiates between the cofactors cyanocobalamin, methylcobalamin, hydroxocobalamin, and adenosylcobalamin. Modified from Buckel (2007). Credit design: L. Montoya and C. F. Franco-Rodríguez.

Coenzyme B_12_ is the only vitamin that contains metal ions; therefore, it is an organometallic cofactor. It also has the most complex structure and largest formula weight, with a chemical structure characterized by a tetrapyrrole with a central Co atom (Figure 1). Cobalamin is derived from the same porphyrin precursor as heme and chlorophyll and the F_420_ coenzyme. It has the most complex structure of any biological cofactor (Bryant et al., 2020) and contains a tetrapyrrole corrin ring surrounding a central Co atom with an oxidation state of 3+. The fifth coordinated position of Co is occupied by a dimethyl-benzimidazole nucleotide loop, and the sixth catalytic upper ligand position is occupied by either a methyl group or deoxyadenosine, leading to methylcobalamin (MeCbl) or deoxyadenosylcobalamin (AdoCbl), respectively (Doxey et al., 2015; Figure 1).

The main driving force for the utilization of Co is the chemistry of this transition metal. The essential ability of Co is to form metal-carbon bonds, which is facilitated by its powerful nucleophilicity. Three cobalt oxidation states participate in the functioning of the B_12_ coenzyme: Co^+^, Co^2+^, and Co^3+^ (Okamoto and Eltis, 2011), each in a coordinated manner and a spatial arrangement facilitated by conformational rearrangements in the corrin ring (Osman et al., 2021).

### 2.2 Biosynthetic pathways for cobalamin

There are essentially two alternative cobalamin biosynthetic routes in bacteria and archaea, aerobic and anaerobic. These routes differ primarily in the contraction stage of the macrocycle: (a) the late insertion pathway is an aerobic pathway because cobalt is inserted into the tetrapyrrole macrocycle after ring contraction, where molecular oxygen is a prerequisite; and (b) the early insertion pathway can operate under anaerobic conditions, where cobalt is chelated before ring contraction (Scott and Roessner, 2002). Despite the differences in the timing of cobalt insertion and the mechanism of ring contraction, many other enzymes associated with methylation and amidations operate in the same order along the pathway (Osman et al., 2021).

In natural ecosystems, B_12_ biosynthesis is energetically expensive and imposes a high metabolic burden on the B_12_ producers. The cobalamin biosynthetic pathway (aerobic or anaerobic) is one of the most complex pathways in nature, requiring approximately 20 enzymes for complete *de novo* synthesis, starting with cobalt (Co^2+^) (Warren et al., 2002). Cofactors are often imported by cobalamin producers, a strategy that is energetically more favorable than biosynthesis (Nijland et al., 2022).

### 2.3 Metabolic processes dependent on cobalamin

Coenzyme B_12_ or cobalamin-dependent enzymes are mainly included in the following groups: (a) transferases (E.C. 2.x.x.x), such as methylcobalamin (MeCbl)-dependent transferases and (b) isomerases (E.C. 5.x.x.x), such as adenosylcobalamin (AdoCbl) deoxyadenosyl cobalamin-dependent isomerase (Table 1). These catalysts are present in both prokaryotes and eukaryotes and reductive dehalogenases (E.C. 1.x.x.x) are found only in organohalide-respiring bacteria (Giedyk et al., 2015).

**Table 1:** Metabolism processes linked to cobalamin are denoted as cobalamin-dependent enzymes (Buckel, 2007; Degnan et al., 2014).

MTR	Methyltetrahydromethanopterin:coenzyme M methyltransferase	2.1.1.86
Mta/Mtt	Methanol/methylamine/trimethylamine methyltransferases	2.1.1.247
Lyases		
PduCDE	Propanediol dehydratase/Glycerol dehydratase	4.2.1.28/4.2.1.30
EutBC	Ethanolamine ammonia-lyase	4.1.3.7
Isomerases		
MCM/SpcA/MutAB	Methylmalonyl-CoA mutase	5.4.99.2
Mgm	2-methyleneglutarate mutase	5.4.99.4
GlmES	Glutamate mutase, metylaspartate mutase	5.4.99.1
IcmAB	Isobutyryl-CoA mutase	5.4.99.13
KamED	b-Lysine 5,6-aminomutase	5.4.3.3
OraSE	D-ornithine 4,5-aminomutase	5.4.3.5

MeCbl serves as the intermediate carrier of activated methyl groups. During the catalytic cycle, the coenzyme shuttles between MeCbl and the highly nucleophilic cobalamin form (Giedyk et al., 2015). Examples of MeCbl-dependent enzymes include methionine synthase, which is a cytosolic methionine synthase (formation of methionine) (Balabanova et al., 2021) that reacts with cobalamin-dependent methionine synthesis and DNA synthesis through the conversion of ribonucleotides to deoxyribonucleotides and tRNA biosynthesis (Banerjee and Ragsdale, 2003).

AdoCbl functions as a source of carbon-based free radicals that are unmasked by homolysis of the cobalt-carbon bond of the coenzyme. Free radicals are subsequently used to remove non-acidic hydrogen atoms from substrates to facilitate a variety of reactions involving cleavage of carbon–carbon, carbon–oxygen, and carbon–nitrogen bonds. Most of these reactions involve the migration of hydroxy-, amino-, and carbon-containing groups. In addition, one class of ribonucleotide reductases uses AdoCbl (Giedyk et al., 2015).

### 2.4 Ecological interactions between cobalamin producers and auxotrophs in marine ecosystems

The natural forms of vitamin B_12_, MeCbl, and AdoCbl are synthesized only by prokaryotes via aerobic and anaerobic pathways (Balabanova et al., 2021). Cyanobacteria and key taxa within α- and γ-Proteobacteria (Sañudo-Wilhelmy et al., 2014), Actinobacteria, and Bacteroidetes Thaumarchaeota (Doxey et al., 2015; Shelton et al., 2019) are included among the cobalamin producers. The involvement of archaea in cobalamin production is poorly understood; thus, the insight into the cobalamin biosynthesis have mainly focused on bacteria (Table 2). The ability to synthesize cobalamin has neither been inherited by eukaryotes nor has it been subjected to lateral gene transfer (Croft et al., 2005). In eukaryotes, vitamin B_12_ is acquired in two ways:

- Directly from a metabolite pool released by prokaryotes, either through cell lysis of the producers or via passive transport (Romine et al., 2017), benefiting cobalamin-dependent eukaryotic consumers, such as the diatom *Thalassiosira pseudonana* (Sultana et al., 2023). Several experimental amendments support this, showing that dissolved cobalamin in the ocean stimulates the growth of eukaryotic phytoplankton (Bonnet et al., 2013) and heterotrophic protists (Wienhausen et al., 2022).
- Indirectly through interactions with prokaryotes, such as mutualism, symbiosis, or predation (Joglar et al., 2021). Among marine cultures, symbiosis and mutualism are the most studied interactions. For example, symbiosis has been explored in co-cultures such as *Ostreococcus tauri* and the alphaproteobacterium *Dinoroseobacter shibae* (Cooper et al., 2018). Additionally, it has been hypothesized that marine sponges obtain cobalamin through symbiotic relationships with associated microbes (Degnan et al., 2014).

**Table 2:** Cobalamin producers in marine ecosystems (Sañudo-Wilhelmy et al., 2014; Wang J. et al., 2024; Wang L. et al., 2024).

Phylum^1^	Order	Described at polymetallic nodules/cobalt crusts
Cyanobacteria	Chroococcales	No
Cyanobacteria	Prochlorales	No
Cyanobacteria	Nostocales	No
Cyanobacteria	Oscillatoriales	No
α-Proteobacteria	*Mycoplana* (Hyphomicrobiales)	No
α-Proteobacteria	Magnetococcales^2^	Yes (Liu et al., 2024)
α-Proteobacteria	*Rhodobacter* (Rhodobacterales)	Yes (Shulga et al., 2022)
β-Proteobacteria	*Achromobacter* (Burkholderiales)	Yes (Zhang et al., 2023)
γ-Proteobacteria^3^	*Pseudomonas* (Pseudomonadales)	Yes (Zhang et al., 2015)
γ-Proteobacteria	*Halomonas* (Oceanospirillales)	Yes (Zhang et al., 2015)
γ-Proteobacteria	*Marinomonas* (Oceanospirillales)	Yes (Zhang et al., 2015)
γ-Proteobacteria	*Neptuniibacter* (Oceanospirillales)	No
Bacteroidetes	*Flavobacterium* (Flavobacteriales)	Yes (Huo et al., 2015)
Actinobacteria	*Micrococcus* (Micrococcales)	Yes (Bergo et al., 2022)
Actinobacteria	*Corynebacterium*, *Nocardia* (Mycobacteriales)	No
Actinobacteria	*Streptomyces* (Kitasatosporales)	No
Firmicutes	*Bacillus* (Caryophanales)	Yes (Liao et al., 2011)
Thaumarchaeota	Nitrosopumilales	Yes (Hollingsworth et al., 2021)
Thaumarchaeota	Nitrososphaerales	Yes (Hollingsworth et al., 2021)

1 Proteobacteria were divided into classes.

2 Magnetococcales have been implicated in the occurrence of biogenic magnetotactic bacteria in polymetallic nodules (Liu et al., 2024).

3 With the exception of Pseudomonas, Halomonas, Marinomonas, and Neptuniibacter, marine γ -Proteobacteria lack genes for B_12_ synthesis (Sañudo-Wilhelmy et al., 2014).

These mechanisms of eukaryotic acquisition of cobalamin from external sources have shaped ecosystems. In all ecosystems, auxotrophs are organisms that depend on cobalamin, but lack the ability to synthesize these cofactors, including both prokaryotes and eukaryotes (Nijland et al., 2022). Examples of auxotrophs are organohalide-respiring bacteria and several eukaryotic lineages, such as vertebrates, most protists, and invertebrates, but not plants, insects, or fungi (Degnan et al., 2014). Intriguingly, higher plants have no cobalamin-dependent enzymes and so do not utilize or synthesize cobalamin (Croft et al., 2006); in comparison, nearly half of marine species are cobalamin auxotrophs (Croft et al., 2005). Eukaryotic auxotrophy has evolved several times, resulting in close and intricate ecological relationships. For example, algae can be influenced by their symbionts, which supply fixed carbon in return for vitamin B_12_ (Grant et al., 2014).

As one of the highly limited nutrients and growth factors controlled by a minority of microbes, B_12_ can be considered as “hard currency” in the global ocean ecosystem (Zhou et al., 2023). Therefore, cobamide sharing creates a network of cobamide-dependent interactions, providing a useful system for studying mechanisms that influence community composition and function (Sokolovskaya et al., 2020). Evidence of this shaping of B vitamins in the photic zone, including vitamin B_12_, has been studied in the Mediterranean Sea and the Eastern Atlantic Ocean, exploring picoplankton and suggesting that cobalamin is crucial for determining the structure and function of microbial ecosystems (Suffridge et al., 2018).

## 3 Cobalt and cobalamin in the water column

### 3.1 Distribution and dynamics of cobalt

Cobalt (Co) is required for the *de novo* synthesis of vitamin B_12_ (Panzeca et al., 2008), as this trace metal serves as the central coordinating ion within the molecule. However, the total dissolved Co concentrations (mainly Co^2+^ and Co^3+^) are relatively low, ranging from 3 to 120 picomolar (pM) in the open ocean (Saito and Moffett, 2002), and only a small fraction of this Co pool is thought to exist in the bioavailable dissolved form as Co^2+^ (Panzeca et al., 2009). Generally, dissolved metals, including Co, increase with depth because of the release associated with the decomposition of organic matter (Gerringa et al., 2021). In comparison, at the surface, dissolved cobalt exhibits low concentrations and high variability, whereas in the ocean interior, cobalt concentrations increase from the surface to intermediate waters and decline in the deep ocean (Tagliabue et al., 2018). Considering the low cobalt concentrations and chemical speciation, the total dissolved cobalt pool of redox state 2+, suitable for B_12_ synthesis, is very low, within the femtomolar (fM) range (Panzeca et al., 2008). This low Co (II) content is consistent with the proportional distribution of dissolved B_12_ in the North Atlantic Ocean according to the abundance of total dissolved Co availability (Panzeca et al., 2009). A similar conclusion was reported by Hawco et al. (2020), who found that Co could limit the growth of the cyanobacterium, *Prochlorococcus*, in the Pacific Ocean.

### 3.2 Cobalamin availability

Vitamin B_12_ is an energetically costly metabolite, which may explain why less than 40% of prokaryotes encode the genes required for its complete biosynthesis (Shelton et al., 2019). In the open ocean, vitamin B_12_ concentrations range from sub-picomolar to picomolar (Sañudo-Wilhelmy et al., 2012). This distribution is influenced by Co availability (Moore et al., 2013), which plays a role in shaping the ecological structure. Sañudo-Wilhelmy et al. (2006) conducted field-based vitamin B_12_ amendment experiments and observed an increase in phytoplankton biomass, leading to the recognition of this metallocofactor as a limiting factor in some marine ecosystems (Wang L. et al., 2024). Additionally, cobalt has been identified as playing a secondary role in biogeochemical interactions, such as phosphorus, whereas nitrogen and iron remain the primary limiting nutrients (Valiela, 2015).

The concentration of vitamin B_12_ in seawater varies significantly across marine regions. For instance, in coastal areas, cobalamin levels range from undetectable to 30 pM (Sañudo-Wilhelmy et al., 2012). Additionally, it has been reported that coastal waters generally contain higher concentrations of vitamin B_12_ compared to open ocean waters (Sañudo-Wilhelmy et al., 2012; Heal et al., 2017). A similar trend was observed when comparing water depths, with lower vitamin B_12_ concentrations found in deeper waters (100–200 m) than in surface and subsurface waters (0–100 m) (Bonnet et al., 2013).

Cobalamin *de novo* synthesis in surface waters has been linked to heterotrophic Proteobacteria bacteria, mainly Rhodobacterales (Gómez-Consarnau et al., 2018), and to photoautotrophic Cyanobacteria synthesizing pseudocobalamin with a peak concentration within the euphotic zone (Heal et al., 2017). These cobalamin providers are related to different biogeographic settings, including the Mediterranean Sea and Eastern Atlantic Ocean (Suffridge et al., 2018), Northwest Atlantic Ocean (Soto et al., 2023), subtropical, equatorial, polar frontal Pacific Ocean (Wienhausen et al., 2022), and North Pacific Ocean (Heal et al., 2017).

Cobalamin compounds (cyanocobalamin, methylcobalamin, and hydroxycobalamin; Figure 1) are labile after aerobic light exposure, even in various aqueous solutions (Vaid et al., 2018). The photosensitivity of cobalamin is attributed to the dissociation of covalent cobalt-carbon bonds upon exposure to light, resulting in its photolabile characteristics (Vaid et al., 2018; Bannon et al., 2024). Consequently, enzymatic reactions involving cobalamin must be conducted under dim light (Giedyk et al., 2015).

## 4 Polymetallic nodules and cobalt crusts: key study sites in cobalt biogeochemistry

### 4.1 Generalities of polymetallic nodules and cobalt crusts

Polymetallic nodules and cobalt-rich crusts are classified in the same paragenetic group (Hazen and Morrison, 2022). Their formation involves complex interactions among geological, chemical, and biological processes that occurr over millions of years (Wang and Müller, 2009). Nodules develop through three main types of precipitation: hydrogenesis refers to the growth of nodules through the direct precipitation of metals from the water column, whereas diagenesis involves the growth of nodules through the precipitation of metals from the sediment pore water. Mixed-type nodules contain layers formed by both hydrogenesis and diagenesis (Benites et al., 2018; Hein et al., 2020). Hydrogenetic nodules form on the sediment surface in well-oxygenated environments where sedimentation rates are low because of the direct supply and precipitation of metallic elements from seawater and diagenetically unenriched sediment pore water (Sujith and Gonsalves, 2021). These nodules are mainly composed of manganese, iron, and trace metals such as nickel (Ni), copper (Cu), cobalt, molybdenum (Mo), and rare earth elements (REE) (Mukhopadhyay et al., 2008). Cobalt crusts are found globally on the ocean floor, generally adjacent to the oxygen minimum zone (OMZ), where less oxidizing conditions prevail (Verlaan and Cronan, 2022). The exposed rock surfaces of the seamounts and ridges are the most concentrated areas. Cobalt-rich crusts occur at shallower depths (<2,500 m) in the deep sea, whereas nodules are formed at much deeper depths (up to 5,500 m) (Verlaan and Cronan, 2022). Initially, it was hypothesized that cobalt precipitates formed solely through inorganic precipitation; however, increasing evidence suggests the involvement of certain bacteria found within nodules and crusts that can oxidize both Mn and Co (Moffett and Ho, 1996; Murray et al., 2007).

### 4.2 Cobalt enrichment in deep-sea minerals

Both polymetallic nodules and cobalt-rich crusts are enriched in cobalt, Ni, Cd, Zn, and REE, relative to their lower concentrations in seawater (Hawco et al., 2018; Hein et al., 2020). Oxidized cobalt (Co^3+^) precipitates in the water column and eventually precipitates via hydrogenesis. Cobalt enrichment in nodules can be up to 100-fold higher than its elemental abundance in Earth’s crust. Cobalt (II) in seawater can undergo surface oxidation to Co (III) via adsorption onto iron and manganese oxyhydroxides (Koschinsky and Hein, 2003; Wang et al., 2009) in minerals, such as birnessite (Wu et al., 2019). Factors such as sedimentation, oxygen concentration, temperature, and growth rate influence Co enrichment in crusts. Slower rates lead to greater Co accumulation, resulting in an average Co content of up to 0.77% in the crust (Verlaan and Cronan, 2022). These factors explain the higher Co concentration in cobalt crusts than in nodules (Mukhopadhyay et al., 2008). Hydrogenetic-type nodules are characterized by higher Co/Mn ratios (Hein et al., 2020). In cobalt-rich crusts, the Co content ranges from 0.3 to 1.2% (Halbach et al., 2017). Nodules from the Clarion-Clipperton Zone (CCZ) have higher Co concentrations, with an average content of 0.21%, compared to other locations (Hein et al., 2020). The Co oxidation state predominantly exists as +3 and a minor fraction as +2 in these deposits (Wegorzewski et al., 2020).

### 4.3 Cobalt crusts: a reliable system for comparative studies

Experiments on cobalt limitation or co-limitation of cobalamin availability have shown that B_12_ synthesis can be restricted by cobalt concentrations in certain ocean regions. For example, amendment experiments conducted in waters with low dissolved cobalt (approximately 20 pM) resulted in a two-fold increase in B_12_ production compared to unamended controls (Panzeca et al., 2008). Furthermore, in amendment experiments conducted in the South Atlantic gyre, the addition of cobalt after nitrogen and iron limitation was alleviated, leading to a significant increase in phytoplankton growth (Browning et al., 2017). It has been proposed that cobalamin availability is regulated not only by photodegradation, alteration, and the supply/demand ratio but also by cobalt availability (Bannon et al., 2022). This suggests that, in surface waters, cobalt and light frequently modulate and limit cobalamin availability, as phytoplankton, especially cyanobacteria, are the main sources of this metallocofactor (Heal et al., 2017). However, this scenario is not equivalent to an increase in depth, considering that anoxic ecosystems favor cobalt dissolution, leading to a long residence time for cobalt (Tagliabue et al., 2018).

Therefore, different conditions of cobalamin availability, such as light, phytoplankton distribution, and oxygen concentration, provoke distinctive ecological networks in the deep sea. In this regard, sites enriched in ferromanganese (Fe-Mn) nodules and cobalt crusts are useful for resolving these ecological questions and testing hypotheses. As mentioned earlier, these deposits are broadly characterized by high levels of Mn oxides, where cobalt is incorporated into the octahedral sheets of phyllomanganates, particularly vernadite (*δ*-MnO_2_) (Wegorzewski et al., 2020). A relevant question arises regarding whether cobalamin synthesizers in these regions play a role in supporting local auxotrophic organisms, or whether and to what extent the communities depend on surface sources, such as cyanobacteria, given that previous studies have shown phytodetritus contributes to particulate organic carbon in sediments within the Clarion Clipperton Zone (Cecchetto et al., 2023).

### 4.4 Thaumarchaeota as potential key players in cobalamin biosynthesis

Thaumarchaeota, formerly known as Crenarchaeota, is a phylum that is frequently found in the deeper waters of the ocean (Heal et al., 2017; Qin et al., 2020). Their distribution is likely due to the sensitivity of ammonia oxidation to light (Merbt et al., 2012). Ammonia-oxidizing archaea (AOA) or Thaumarchaeota strains are typically isolated under dark conditions (Stieglmeier et al., 2014). Indeed, the initial steps of both ammonia-oxidizing pathways, archaeal and bacterial (AOA and AOB, respectively), are inhibited by light (Merbt et al., 2012). In this sense, ammonia-oxidizing archaea are abundant in sediments (coastal and estuarine) (Francis et al., 2005). These archaea are the major cobalamin sources in the oxygen-deficient zone (ODZ) of coastal productive ecosystems (Heal et al., 2017), and as suggested by their genomic potential, they could potentially inhabit bathypelagic habitats (Doxey et al., 2015). Interestingly, Thaumarchaeota, formerly known as Crenarchaeota, is a significant source of vitamin B_12_ in these environments (Heal et al., 2017).

Isolation of the non-cyanobacterial and cobalamin synthesizers *Nitrosopumilus* spp. (Thaumarchaeota), *Sulfitobacter* sp. SA11, and *Ruegeria pomeroyi* DSS-3 (α-Proteobacteria) from deep water support the hypothesis of vertical niche differentiation (Heal et al., 2017). This is particularly noteworthy given that cobalamin itself is photosensitive (Bannon et al., 2024), suggesting an interplay between the light environment and cobalamin dynamics in the ocean. The growth of Thaumarchaeota, also known as AOA, can be promoted by ammonia provided by auxotrophs (Heal et al., 2017; Hollibaugh, 2017). Therefore, in mesophotic ecosystems, only selected cobalamin auxotrophic hosts (bacteria and eukaryotes) provide this essential nutrient for AOA. This contrasts with Cyanobacteria, which have a distinct euphotic ecological niche.

**Figure 2:**
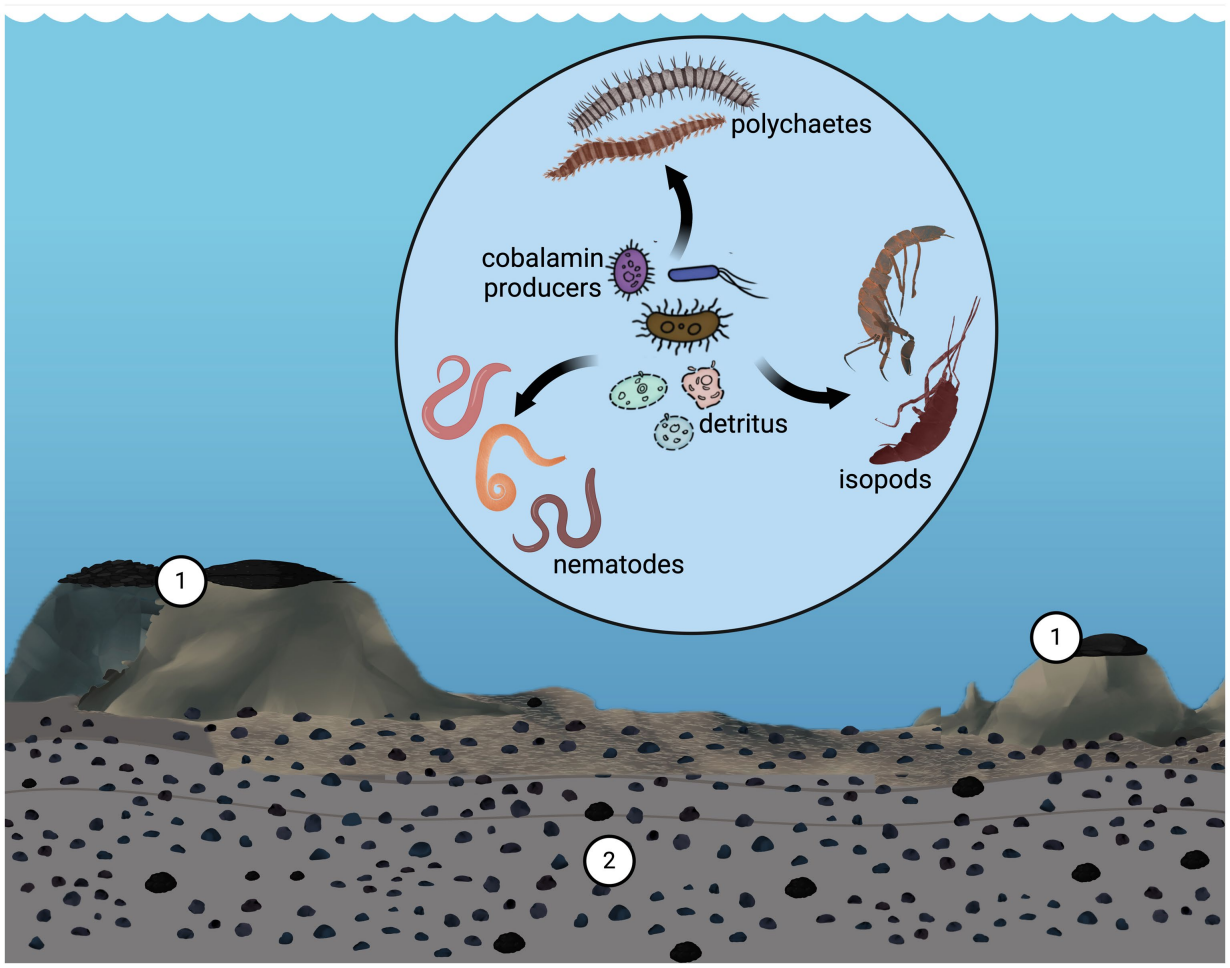
Scheme illustrates the proposed interrelationship between cobalt found in cobalt crusts (1) and polymetallic nodules (2), and cobalamin and trophic interactions within the community in these systems, mediated through synthesizers (prokaryotes) and bacterivores. Credit design: L. Montoya and C. F. Franco-Rodríguez.

The presence of the cobalamin biosynthetic pathway in an ecosystem implies a level of sustainability at which auxotrophic organisms can thrive only in communities that provide this essential vitamin. In this context, we hypothesized that certain taxa characteristic of these aquatic environments play key roles in cobalamin biosynthesis. In this regard, we highlight the abundance of Thaumarchaeota associated with these concretions, as recognized by the clone library (Shulse et al., 2017) and metagenomic approaches (Zhang et al., 2023). Notably, analysis of bacterial and archaeal 16S rRNA gene sequences from deep-sea polymetallic nodules and sediment extracted from the South Pacific Gyre revealed that the phylum Thaumarchaeota was more abundant in the nodules than in the surrounding sediments (Shiraishi et al., 2016). Based on this, we suggest that members of this archaeal phylum may serve as a source of cobalamin for both prokaryotes and eukaryotes. Furthermore, considering the high cobalt content in cobalt crusts and some polymetallic nodules, it is plausible that, in these habitats, a cobalt-cobalamin connection exists along the food web and involves prokaryotes and metazoans (Figure 2).

Therefore, cobalamin-producing prokaryotes, including Thaumarchaeota, play key roles in linking cobalt and cobalamin. Furthermore, the availability of cobalt, a critical element in the organometallic metabolite cobalamin, may explain the sustainability of auxotrophs in these deep-sea regions. To examine this claim, it is essential to study organisms, such as Thaumarchaeota and other cobalamin producers, as they provide this vital metabolite to auxotrophic bacteria and metazoans. This study aimed to explore the relative abundance of these prokaryotes, and the genetic repertoire associated with cobalamin synthesis in the metazoan microbiome thriving on cobalt crusts and polymetallic nodules and compare them with other deep-sea regions. The relationship between metazoans and their cobalamin-producing symbionts or mutualists may be particularly relevant for bacterivorous meiofauna.

### 4.5 Proposed ecological networks driven by cobalt and cobalamin availability

Prokaryotic B_12_ sources in some unicellular eukaryotes and metazoans may occur through symbiosis, predation, or commensalism. Within the nodule and cobalt crust communities, some prokaryotes, such as α- and γ-Proteobacteria and Thaumarchaeota, may benefit to other populations with essential vitamin B_12_. An ecological example to argue this is the commensalism between the auxotrophic algal species *Porphyridium purpureum*, which obtains cobalamin from marine *Halomonas* sp. In return, algae provide γ-Proteobacteria with carbon sources (Croft et al., 2005) and and also acquire cobalamin through particulate organic carbon (POC) and dissolved organic carbon (DOC) (Moran et al., 2022). The implications of cobalt availability modulation on cobalamin synthesis have been demonstrated in surface water samples from the North Atlantic Ocean (Panzeca et al., 2008). Therefore, we propose that contrary to the cobalt limitation prevailing in the North Atlantic Ocean, as reported by Panzeca et al. (2008), cobalt crusts and polymetallic nodules support an abundance of cobalamin synthesizers in the absence of light.

Polymetallic nodules provide crucial hard surfaces for deep-sea benthic life with organisms such as stalked and encrusting sponges (Stratmann et al., 2021). An example is *Plenaster craigi*, an abundant sponge frequently attached to nodules that relies on particulate organic matter, bacteria, and other microbes (Taboada et al., 2018). Some annelids like the polychaeta *Neanthes goodayi* reside inside polymetallic nodules highlight the importance of the mineral itself as microhabitat (Drennan et al., 2021; Neal et al., 2022). In this sense, nodules and crusts represent a cobalt micro-oasis to sessile life that is likely to provide metal content that is beneficial to the synthesis of cobalamine. The experimental extraction of nodules from the Clarion-Clipperton Fracture Zone (CCZ) in the central Pacific has recorded impacts on biodiversity loss and changes in deep-sea water-sediment processes (Bonifácio et al., 2024; Stratmann, 2023; Stratmann et al., 2021). Bacterivores, such as Bryozoa, Cnidaria, Platyhelminthes, and Porifera, are among the most affected ecological groups (Stratmann et al., 2021). Other studies have experimentally focused on Nematoda and found that this taxon does not recover after extraction (Miljutin et al., 2011). These taxa, together with the rotifers and polychaetes present in nodules and crusts, represent a trophic link between bacteria and larger fauna (Stratmann, 2023; Uhlenkott et al., 2023). In this context, we propose a role for cobalt in the loss of taxa induced by nodule extraction. This proposal arises from keeping in mind that model species for some of the phyla described in the CCZ metazoan organisms acquire vitamin B_12_ from bacteria directly by ingestion or commensalism, as cobalamin-producing bacteria are abundant resident gut microbes (Degnan et al., 2014), such as α-, γ-Proteobacteria and Thaumarchaeota (Shulse et al., 2017). Nematodes are noteworthy in this context as they are the most abundant meiobenthic taxon in the ecosystems of nodule-bearing deep-sea sediments (Hauquier et al., 2019). Experimental work with the laboratory nematode *Caenorhabditis elegans* highlights cobalamin limitation, resulting in reduced fertility and longevity (Degnan et al., 2014) and predatory behavior, such as *Pristionchus pacificus*, where vitamin B_12_ is an important inducer (Lo and Sommer, 2022). Additional experimental work has shown symbiotic synthesis of cobalamin in a microbial co-culture of the bacteria *Colwellia* sp. and *Roseovarius* sp. (Wienhausen et al., 2024). Shallow water *Aplysina aerophoba* sponges show B_12_ synthesis dependency on Poribacteria (Siegl et al., 2011). Similar ecological interactions can occur in the mesophotic water columns and seafloor ecosystems.

## 5 Future research questions for understanding cobalt-cobalamin dynamics in deep-sea ecosystems

The ecological significance of cobalamin (vitamin B_12_) in other ecosystems remains largely unexplored. Future field studies should clarify the physiological requirements of organisms for cobalamin, particularly the minimum concentration required for growth and survival in the absence of light and oxygen. Laboratory evidence demonstrating the effects of B vitamins, specifically cobalamin, on microplankton has shed light on how marine bacteria fulfill the cobalamin requirements of auxotrophic organisms. For example, key research, such as the co-culture of Ostreococcus tauri with the bacterium *Dinoroseobacter shibae* (Cooper et al., 2018), provides a foundation for analyzing cobalt-rich crust prokaryotic and eukaryotic isolates. In addition, the co-culture approach employed by Wienhausen et al. (2024) further enhances research on prokaryote-prokaryote interactions, revealing a closer ecological connection where a symbiosis of two bacteria (Colwellia sp. and Roseovarius sp.) is required for complete cobalamin synthesis.

It is crucial to determine whether metazoans living in the absence of light selectively or preferentially ingest cobalamin directly or rely on symbiotic microbes (endo- or exosymbionts) to synthesize and provide cobalamin. Furthermore, investigating whether cobalamin-dependent metabolism offers an advantage to organisms living in environments with high concentrations of cobalt despite the absence of light or oxygen is essential for understanding the occurrence of microbial species that use cobalt and their adaptations.

The potential influence of cobalt availability on deep-sea metazoan distribution owing to cobalamin requirements highlights the urgent need for further research on the ecological implications of cobalt. In this regard, cobalt crusts and nodule habitats are sites for study. Advancements in genomics and metagenomics, particularly regarding the microbiome of metazoans, will be instrumental in characterizing their potential role as vitamin B12 auxotrophs. A valuable strategy for understanding the importance of cobalamin in ocean microbial communities and their integration along the food web will lead to new discoveries (Wang L. et al., 2024).

However, the chemical profile of Co in areas containing Co crusts and polymetallic nodules remains poorly characterized. A thorough investigation of cobalt distribution, particularly its redox speciation, is needed, as is research on abiotic and biotic cobalt oxidation from Co^2+^ to Co^3+^ within these systems, where microorganisms interact with the global cobalt biogeochemical cycle (Swanner et al., 2014). Some approaches already used in this context have involved cobalt amendment experiments (Bannon et al., 2022) and characterization of cobalamin-dependent enzymes (Hawco et al., 2020). This review provides a synthesis for future research determining whether cobalt limits or co-limits ecosystems in the absence of light and/or oxygen.

Studies focused on this topic will not only provide a clearer understanding of this subset of prokaryotes in the global ocean but will also shed light on its function (Zhou et al., 2023). As stated by Moore et al. (2013) and Giovannelli (2023), the distribution and availability of trace elements in the environment are required to understand the diversity of life on the planet.

## Data Availability

The original contributions presented in the study are included in the article/supplementary material, further inquiries can be directed to the corresponding authors.
